# Machine-Learning-Based m5C Score for the Prognosis Diagnosis of Osteosarcoma

**DOI:** 10.1155/2021/1629318

**Published:** 2021-10-11

**Authors:** Haijie Zhang, Peipei Xu, Yichang Song

**Affiliations:** ^1^The Eighth Affiliated Hospital of Sun Yat-Sen University, Shenzhen 518033, China; ^2^PET/CT Center, The First Affiliated Hospital of Shenzhen University, Shenzhen 518000, China

## Abstract

**Background:**

Osteosarcoma is a common and highly metastatic malignant tumor, and m5C RNA methylation regulates various biological processes. The purpose of this study was to explore the prognostic role of m5C in osteosarcoma using machine learning.

**Methods:**

Osteosarcoma gene data and the corresponding clinical information were downloaded from the GEO database. Machine learning methods were used to screen m5C-related genes and construct m5C scores. In addition, the clusterProfiler package was used to predict the m5C-related functional pathways. xCell and CIBERSORT were used to calculate the immune microenvironment cells. GSVA was applied to analyze different categories of m5C genes, and the correlation between the GSVA and m5C scores was evaluated.

**Results:**

Twenty m5C genes were identified, and 54 related genes were screened. The m5C score was constructed based on the PCA score. With an increase in the m5C score, the expression of m5C genes and their related genes changed. Functional analysis indicated that the focal adhesion, cell-substrate adherens junction, cell adhesion molecule binding, and E2F targets might change with the m5C score. The naive B cells and CD4^+^ memory T cell also changed with the m5C score. The results of the correlation analysis showed that the m5C score was significantly correlated with the reader and eraser genes.

**Conclusion:**

The m5C score might be a prognostic index for osteosarcoma.

## 1. Introduction

Osteosarcoma is a common primary bone malignancy with a high rate of incidence in children and adolescents [1, 2]. Osteosarcoma is highly aggressive, is metastatic, and has a high risk of recurrence after treatment [3]. Currently, the main treatment methods for patients with osteosarcoma include surgery, radiotherapy, chemotherapy, and combination therapies [4, 5]. Osteosarcoma is thought to arise from osteoblasts in rapidly growing bones [6]. Changes in cellular heterogeneity and immune dynamics lead to complex molecular and genetic mechanisms that make conventional treatment regimens less effective in a clinical setting [7, 8]. Studies have shown that the estimated survival rate of patients with metastatic osteosarcoma undergoing routine treatment is less than 5 years [9]. Therefore, a clear diagnosis and precisely targeted therapy are significant for patients with osteosarcoma. The new generations of sequencing technology and data analysis methods provide an efficient and convenient technical auxiliary means for the exploration of therapeutic targets for osteosarcoma.

As a branch of computer science and statistics [10], machine learning generates predictive models mainly by learning from training data. Its application in the field of medicine has attracted significant attention, including the diagnosis, prognosis, and treatment cycle prediction of the disease. The literature has shown that machine learning methods have been applied in drug discovery [11], the management of hematologic malignancies [12], and epilepsy pathology monitoring [13].

RNA methylation is involved in the regulation of various biological processes, and its dysregulation is closely related to the occurrence of human malignant tumors [14]. In addition, 5-methylcytosine (m5C) is a type of RNA methylation modification located in the untranslated region of the mRNA transcript [15]. M5C is involved in various RNA metabolisms, including mRNA output, RNA stabilization, and translation, through the dynamic regulation of a series of important mediator proteins (writer, eraser, and reader) [16]. Studies have shown that m5C plays an important role in the diagnosis and prognosis of cancer, including hepatocellular carcinoma [17], squamous cell carcinoma of the head and neck [18], lung adenocarcinoma [19], triple-negative breast cancer [20], and gastrointestinal cancer [21]. However, the role of m5C in osteosarcoma remains unclear.

The prognostic role of the m5C score in osteosarcoma is currently unknown. In this study, we analyzed the data in GEO. A machine learning method was used to screen and construct the m5C score. Related functional pathways were predicted. The correlation between clinical factors, m5C scores, and the prognosis was analyzed. These results provide a scientific basis for using the m5C score as a prognostic indicator for patients with osteosarcoma.

## 2. Methods

### 2.1. Datasets and Data Preprocessing

The GSE21257 and GSE39058 gene expression profile and corresponding clinical information were downloaded from the GEO database (https://www.ncbi.nlm.nih.gov/gds/), with the GSE39058 dataset as the validation set. The microarray data of the GPL10295 platform (Illumina human-6 v2.0 expression beadchip) include RNA expression and clinical characteristics (e.g., age, sex, grade, metastasis, and pathology) from 53 patients with osteosarcoma. Microarray data probes were annotated based on the gene names, and the mean value of all probes with the same gene name was obtained. All data were then normalized.

### 2.2. Establishment of the m5C Score

The 21 m5C genes, including the synonym of these genes, were intersected with the osteosarcoma dataset (GPL10295), leaving 20 genes (i.e., DNMT1, DNMT3A, DNMT3B, MBD1, MBD2, MBD3, MBD4, MECP2, NEIL1, NTHL1, SMUG1, TDG, UHRF1, UHRF2, UNG, ZBTB33, ZBTB4, TET1, TET2, and TET3). Next, a correlation analysis was conducted among these 20 genes, and genes with related coefficient >0.6, with *p* < 0.05, were considered as related genes. The univariate analysis was used for the related gene dimensionality reduction. After the random survival forest and PCA were performed, the risk score model was constructed as follows: m5C score=∑*PC*1_*A*_ − ∑*PC*1_*B*_, where *A* represents the gene with HR > 1 and *B* represents the gene with HR < 1. The m5C score was verified in GSE21257 and GSE39058. ROC curves were performed to diagnose the effectiveness of the model. After three main classifications (reader, eraser, and writer) of the m5C gene and the complete gene set were analyzed using GSVA, the correlation analysis was performed between them and the m5C score.

### 2.3. Functional Annotation Analysis

An analysis of the difference between the two groups with high- and low-risk scores in the expression matrix was conducted, and the standard was set as abs (logFC) > log2 (1.5), with *p* value <0.05. A gene ontology (GO) analysis was conducted for these differentially expressed genes, including cellular component (CC), molecular function (MF), and biological process (BP) analyses. The limit for a significant enrichment was set at *p* < 0.05. For the correlation analysis of all genes in the expression matrix of this gene, the R package clusterProfiler [22] was used to conduct a gene set enrichment analysis (GSEA). The criteria of statistical significance were set as |NES| >1, with NOM *p* value <0.05 and an FDR *q* value < 0.25. The clusterProfiler package was then applied to evaluate and analyze the GO biological process, KEGG pathway, and HALLMARK pathway of the genes.

### 2.4. Immune Cell Infiltration Analysis

In this study, xCell [23] and CIBERSORT were used to calculate immune microenvironment cells. The method integrates the benefits of a gene enrichment analysis through a deconvolution to evaluate 64 cell types involving multiple adaptive and innate immune cells, hematopoietic progenitor cells, epithelial cells, and extracellular matrix cells. These included 48 microenvironment-related tumor cells.

### 2.5. Statistical Analysis

To analyze differentially expressed genes, we used the Benjamini–Hochberg method, which converts *p* values into FDR to identify important genes. All survival curves were generated and visualized using the R package survminer and a Kaplan–Meier analysis. The statistical significance of the differences was determined using the log-rank test in each dataset. A pheatmap was adopted to generate heatmaps. R (https://www.r-project.org/, version 3.6.1) was used to conduct a statistical analysis. The data were visualized using the R package ggplot2. After the Shapiro–Wilk normality test was used to check the normality of the variables, the differences between the two groups were compared using unpaired Student's *t*-test with the normally distributed variables, and the Wilcoxon test was conducted to compare the variables without normally distributed. The statistical significance was set at *p* < 0.05.

## 3. Results

### 3.1. Establishment of the m5C Score for Osteosarcoma

**Figure 1:**
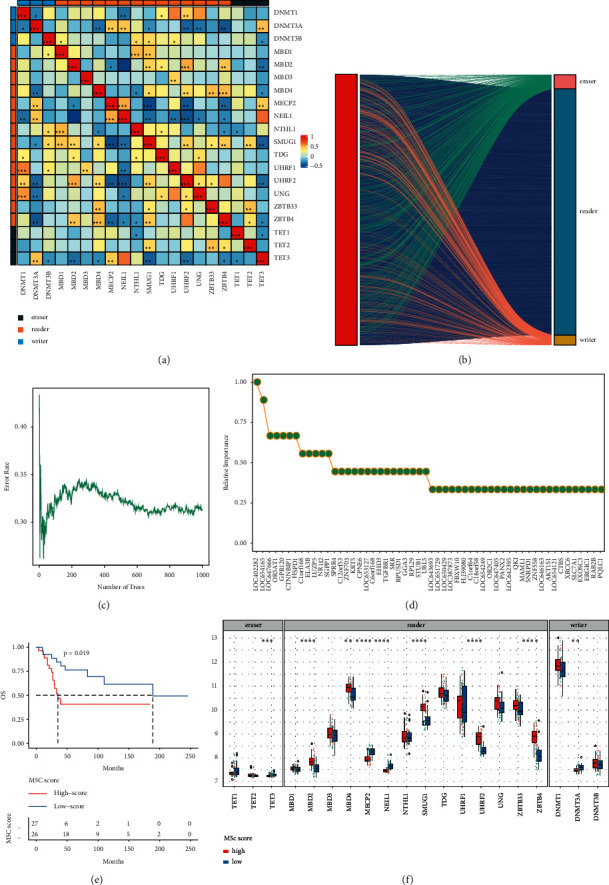
Construction of the m5C risk model. (a) Correlation analysis results of m5C genes. (b) The relationship between the m5C gene and its related genes. (c) Error rate as a function of the classification tree. (d) Related importance values for the genes. (e) Overall survival in the high and low m5C score group. (f) The expression level of the m5C gene under high and low m5C scores. ^∗^ indicates significance with *p* < 0.05.

**Figure 2:**
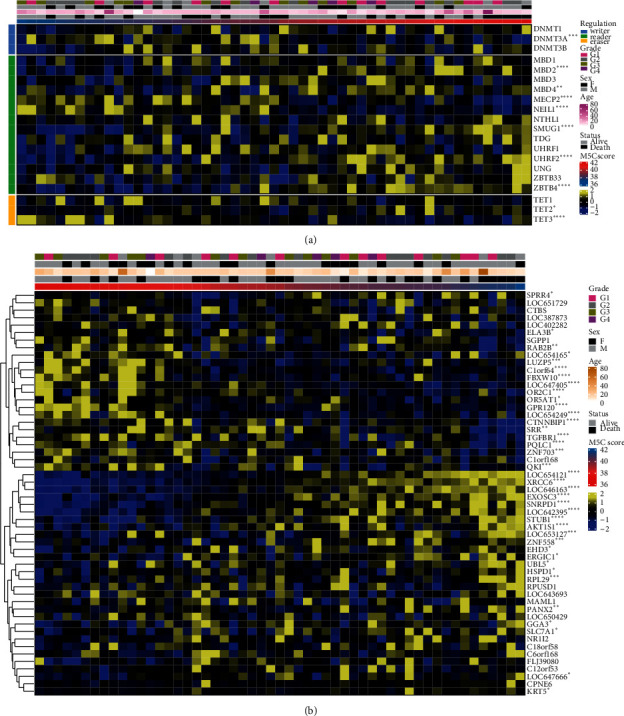
M5C score and gene expression. (a) The expression of m5C genes. (b) The expression of constructing PCA score genes.

To establish the m5C score, we obtained m5C gene data from the GEO database and screened 20 m5C genes. Their survival analysis in GSE21257 was conducted independently (Figures S1 and S2). Correlation analysis among the 20 m5C genes in the osteosarcoma dataset indicated a certain amount of interrelationship among the m5C genes (Figure 1(a)). Based on 20 m5C genes, a gene set containing 3,008 genes was constructed (Figure 1(b)). A total of 1,929 genes were screened out using univariate analysis, and 54 genes were identified in the random survival forest (Figures 1(c) and 1(d)). After a univariate analysis (Figure S3) and PCA scoring of these genes, the m5C score was calculated. A survival analysis showed that patients with a high-risk score had a poor prognosis (*p* value less than 0.05) (Figure 1(e)). The risk score was verified in the GSE39058 dataset (Figure S4). ROC curves were performed to diagnose the effectiveness of the model. The AUC value of our model was higher than that of others [24] (Figure S5). The genes were divided into high and low groups based on their risk scores. The expression of m5C differed, including TET3, MBD2, UHRF2, MECP2, ZBTB4, and SMUG1 (Figure 1(f)). With an increase in the risk score, the expression of the m5C gene changed, including DNMT3A, MBD2, MECP2, NEIL1, and SMUG1 (Figure 2(a)). As the risk score increased, the expression levels of the constructed PCA score genes changed, including C1orf64, FBXW10, LOC647405, OR2C1, AKT1S1, XRCC6, EXOSC3, and SNRPD1 (Figure 2(b)).

### 3.2. M5C Score-Related Functional Analysis of Osteosarcoma

**Figure 3:**
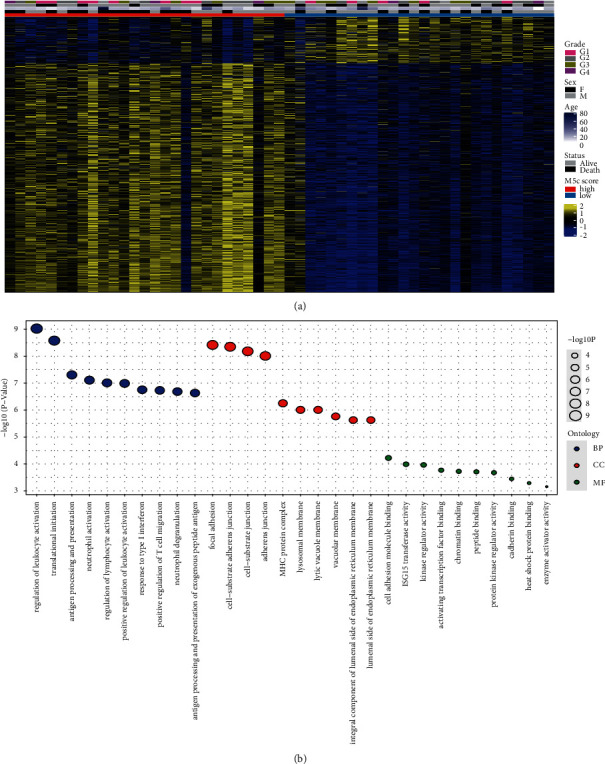
Differential genes and functional analysis between high and low m5C score groups. (a) The expression of differential genes. (b) Functional analysis of differential genes.

**Figure 4:**
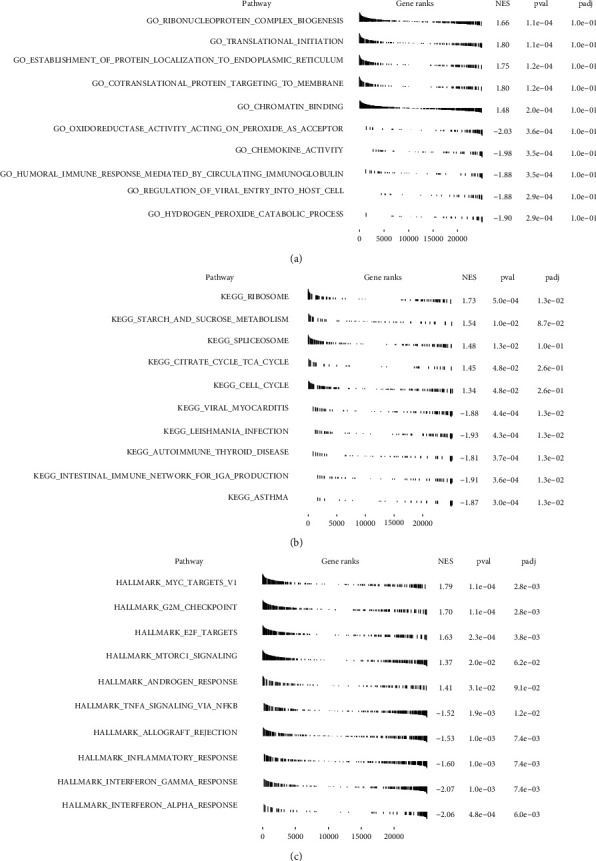
GSEA based on the m5C score in osteosarcoma. (a) GO enrichment analysis. (b) KEGG pathway analysis. (c) HALLMARK analysis.

Next, we assessed the expression of genes in the high and low groups based on the m5C scores (Figure 3(a)). There were conspicuous differences in the gene expression between the high and low m5C score groups. A functional analysis was conducted on the differentially expressed genes, including BP, CC, and MF (Figure 3(b)). Among them, with BP, the regulation of leukocyte activation and translational initiation was significant. With CC, the focal adhesion, cell-substrate adherens junction, cell-substrate junction, and adherens junction were significant. With MF, the cell adhesion of the molecule binding, ISG15 transferase activity, and kinase regulator activity were significant. Meanwhile, we conducted a GSEA based on the m5C score, including GO enrichment (Figure 4(a)), KEGG pathway (Figure 4(b)), and HALLMARK (Figure 4(c)) analyses. The GO analysis results showed that the ribonucleoprotein complex biogenesis, translational initiation, and application of protein localization to endoplasmic reticulum pathways were significant. A KEGG analysis showed that the ribosome, starch and sucrose metabolism, and spliceosome pathways were significant. The results of the HALLMARK analysis showed that the MYC target V1, the G2M checkpoint, and the E2F target pathways were significant.

### 3.3. Immune Infiltration Analysis for the m5C Score

**Figure 5:**
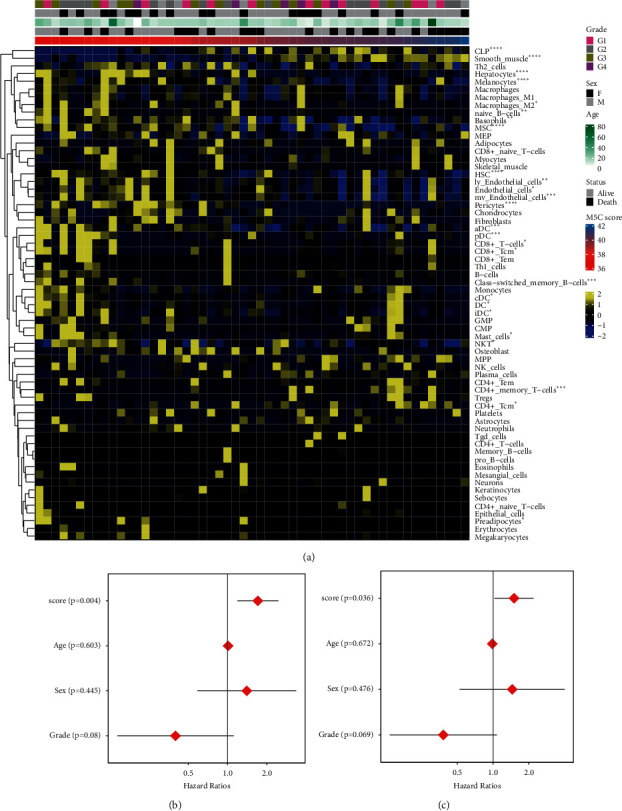
Immune cell infiltration analysis and prognosis analysis for the m5C score. (a) Immune cell infiltration analysis. (b) Univariate analysis. (c) Multivariate analysis.

Next, xCell (Figure 5(a)) and CIBERSORT (Figure S6) were used to analyze the immune cell infiltration. As the m5C score increased, the expression of immune cell infiltrates changed, including the CLP, smooth muscle, hepatocytes, melanocytes, MSCs, and HSCs.

### 3.4. M5C Score as an Independent Prognostic Factor in Osteosarcoma Patients

To further validate the prognostic value of m5C scores, the clinical factors and m5C scores of patients with osteosarcoma were evaluated. The results of a univariate Cox regression analysis (Figure 5(b)) and a multivariate Cox regression analysis (Figure 5(c)) showed that the *p* values of the age, sex, and grade were all greater than 0.05. These findings suggested that these clinical factors in patients are indistinctively associated with the prognosis. The m5C score was significantly correlated with patient prognosis, with *p*=0.004 and *p*=0.036, respectively. Therefore, the m5C score might be an independent prognostic factor in patients with osteosarcoma.

### 3.5. Relationship between the m5C Score and m5C in Osteosarcoma

**Figure 6:**
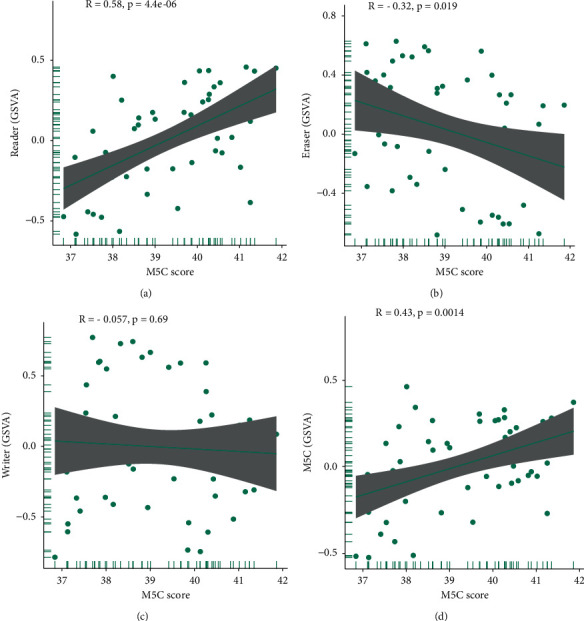
Correlation analysis between the m5C score and m5C gene. Correlation analysis of the m5C genes and the m5C scores, including (a) the reader score, (b) eraser score, (c) writer score, and (d) m5C gene set score.

To further explore the relationship between the m5C gene score and m5C score, we conducted a GSVA on the three major classifications of the m5C gene (reader, eraser, and writer) and the complete m5C gene set. A correlation analysis was applied using the m5C score and the above reader (Figure 6(a)), eraser (Figure 6(b)), and writer (Figure 6(c)) GSVA scores, as well as the GSVA score of the m5C gene set (Figure 6(d)). The GSVA score of the reader was positively correlated with the m5C score, whereas the eraser score was negatively correlated. There was no significant correlation between the GSVA score of the writer and m5C scores. The m5C gene set score was positively correlated with m5C. Thus, there might be a relationship between the m5C scores and m5C genes in osteosarcoma.

## 4. Discussion

Although some studies have suggested that an m5C RNA modification is associated with the genesis and progression of cancer [25], the underlying relationship between osteosarcoma and m5C remains unclear. In this study, we established a risk model based on the m5C scores. Compared with other models [24], the m5C score has higher diagnostic efficiency. Furthermore, the m5C score can be used as an independent prognostic factor in patients with osteosarcoma.

The m5C score, including C1orf64, AKT1S1, and XRCC6, was constructed in this study. Among them, C1orf64 can be used as a protective factor in patients with osteosarcoma, and its expression decreases with an increase in the m5C score. SRARP (C1orf64) is a tumor suppressor that can be used to predict the clinical outcomes of malignant tumors [26]. In addition, AKT1S1 and XRCC6 were identified as risk factors. A high expression of AKT1S1 is positively correlated with a poor prognosis in liver cancer patients [27]. In addition, XRCC6 is involved in the poor prognosis of human osteosarcoma cells [28] and prostate cancer [29]. All of these demonstrated the reliability of predicting the prognosis of patients based on their m5C score. Regardless, how the genes constructing the m5C score regulate the development of osteosarcoma still requires further research and exploration.

M5C regulates the transcriptome expression mainly through the dynamic regulation of methyltransferases (writers), binding proteins (readers), and demethylases (eraser) [30]. The eraser, reader, and writer can change significantly with the change in the m5C score, including MBD2, UHRF2, ZBTB4, and TET3. Among them, MBD2 inhibits DNA methylation and activates prometastatic genes [31, 32]. In 5-azaCdR-induced breast cancer, MBD2 depletion can inhibit cell invasion [33]. It has been reported that UHRF2 was associated with cell invasion, migration, and lymphatic metastasis of intrahepatic cholangiocarcinoma [34]. TET3 inhibits ovarian cancer by blocking TGF-*β*1-induced EMT [35]. These results suggested that MBD2, UHRF2, and TET3 may be involved in the process of tumor metastasis. Our functional analysis also demonstrates this point. Focal adhesion was significantly increased in terms of the m5C score and high/low m5C score groups, including the focal adhesion, cell-substrate adherens junction, cell adhesion molecule binding, and E2F targets. There is a relationship between cell adhesion and cancer metabolism [36], and it has been shown in the literature that cell adhesion molecules play an important role in the development of cancer and are clinical markers for the effectiveness of cancer therapy [37]. E2F dysfunction may contribute to cancer development [38]. Regulation of the PRMT5-E2F1 axis can promote the migration and invasion of tumor cells [39]. Thus, m5C might influence cancer development by regulating the functional downstream pathways. These results further proved that the m5C score has a certain prognostic role in patients with osteosarcoma.

However, in our analysis, the expression of ZBTB4 increased with an increase in the m5C score. In colorectal cancer, patients with a high ZBTB4 expression have a better prognosis [40]. This is inconsistent with our analysis. We speculated that this might be due to the complexity of the biological functions of cells and the complexity of the microenvironment of a tumor.

Tumor development is closely related to immune invasion [41, 42]. With the change in the m5C score, the immune cells also changed significantly, including naive B cells and CD4^+^ memory T cells. Naive B cells are involved in the prognosis of hepatocellular carcinoma and colorectal cancer [43, 44]. A high abundance of B-cell infiltration could have a good prognostic effect. The study showed that CD4^+^ follicular helper T-cell infiltration could be used to predict breast cancer survival [45]. The resistance of pancreatic cancer cells is also associated with changes in CD4^+^ memory T cells [46]. Therefore, we speculated that m5C could affect the prognosis of osteosarcoma by regulating the infiltration of immune cells.

Combined with the above analysis, we believe that the m5C score can be used as a prognostic index for osteosarcoma. The specific mechanism by which m5C regulates the osteosarcoma prognosis requires further exploration and specific experimental verification.

## 5. Conclusion

In this study, patients with high m5C scores had a poor prognosis. The m5C score could influence cellular changes in the functional regulation and immune microenvironment related to osteosarcoma metastases. In conclusion, the m5C score could be used as an independent prognostic factor for the diagnosis of osteosarcoma.

## Data Availability

The microarray data and clinical data we used in this research were downloaded from the GEO database (https://www.ncbi.nlm.nih.gov/geo/query/acc.cgi?acc=GSE21257; https://www.ncbi.nlm.nih.gov/geo/query/acc.cgi?acc=GSE39058; https://www.ncbi.nlm.nih.gov/geo/query/acc.cgi?acc=GPL10295).

## Supplementary Materials

Supplementary Materials Figures S1 and S2: the survival analysis of DNMT1, DNMT3A, DNMT3B, MBD1, MBD2, MBD3, MBD4, MECP2, NEIL1, NTHL1, SMUG1, TDG, TET1, TET2, TET3, UHRF1, UHRF2, UNG, ZBTB4, and ZBTB33. Figure S3: the univariate analysis results of 54 genes. Univariate analysis showed that these 54 m5C-related genes might affect the survival time of the patients. Figure S4: the survival analysis of the m5C score in GSE39058. Figure S5: the ROC curves of our model (A) and others (B). Figure S6: the immune cell infiltration analysis by the CIBERSORT algorithm.
