# Supplementary figures and images for: Machine-Learning-Based m5C Score for the Prognosis Diagnosis of Osteosarcoma
Source: J Oncol. 2021 Oct 11;2021:1629318. doi: 10.1155/2021/1629318 (PMC8523252; doi:10.1155/2021/1629318)

## The univariate analysis results of 54 genes

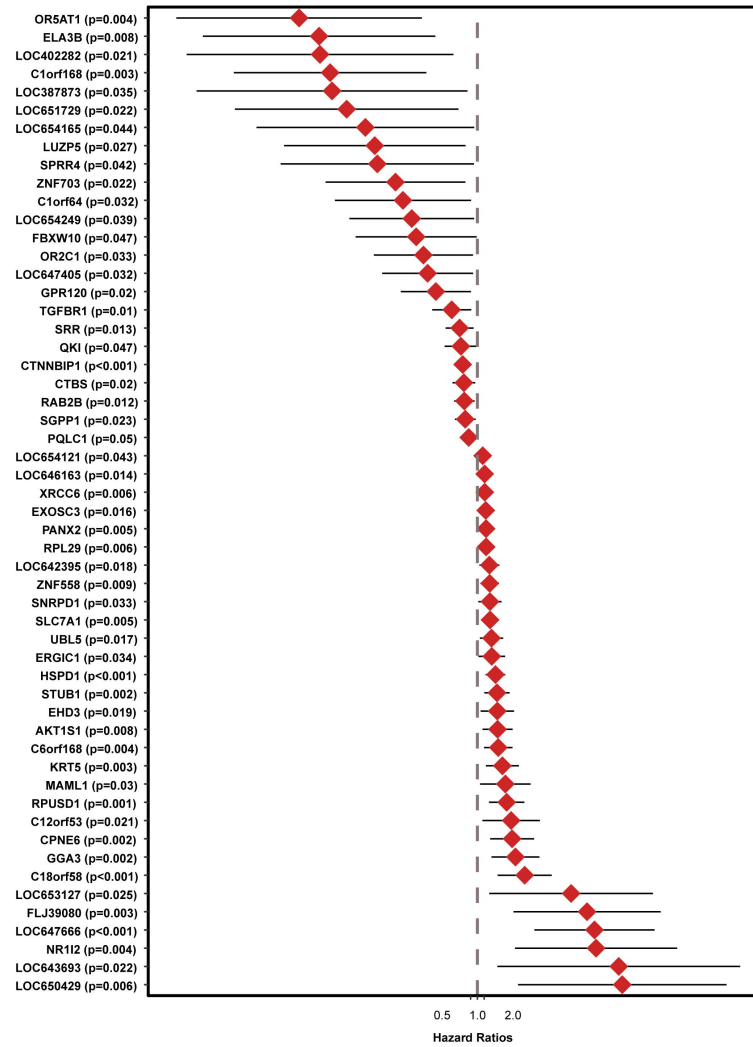

Figure S3: The results of univariate analysis.

Supplement: Supplementary Materials — Figures S1 and S2: the survival analysis of DNMT1, DNMT3A, DNMT3B, MBD1, MBD2, MBD3, MBD4, MECP2, NEIL1, NTHL1, SMUG1, TDG, TET1, TET2, TET3, UHRF1, UHRF2, UNG, ZBTB4, and ZBTB33. Figure S3: the univariate analysis results of 54 genes. Univariate analysis showed that these 54 m5C-related genes might affect the survival time of the patients. Figure S4: the survival analysis of the m5C score in GSE39058. Figure S5: the ROC curves of our model (A) and others (B). Figure S6: the immune cell infiltration analysis by the CIBERSORT algorithm. [file 1629318.f1.zip › 1629318.f1/Figure S3.pdf]

## The survival analysis of m5C score in GSE39058

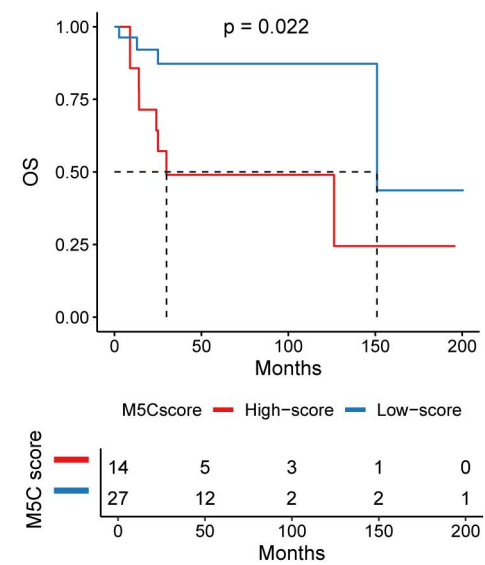

Figure S4: The survival analysis.

Supplement: Supplementary Materials — Figures S1 and S2: the survival analysis of DNMT1, DNMT3A, DNMT3B, MBD1, MBD2, MBD3, MBD4, MECP2, NEIL1, NTHL1, SMUG1, TDG, TET1, TET2, TET3, UHRF1, UHRF2, UNG, ZBTB4, and ZBTB33. Figure S3: the univariate analysis results of 54 genes. Univariate analysis showed that these 54 m5C-related genes might affect the survival time of the patients. Figure S4: the survival analysis of the m5C score in GSE39058. Figure S5: the ROC curves of our model (A) and others (B). Figure S6: the immune cell infiltration analysis by the CIBERSORT algorithm. [file 1629318.f1.zip › 1629318.f1/Figure S4.pdf]

## The ROC curves of our model and other

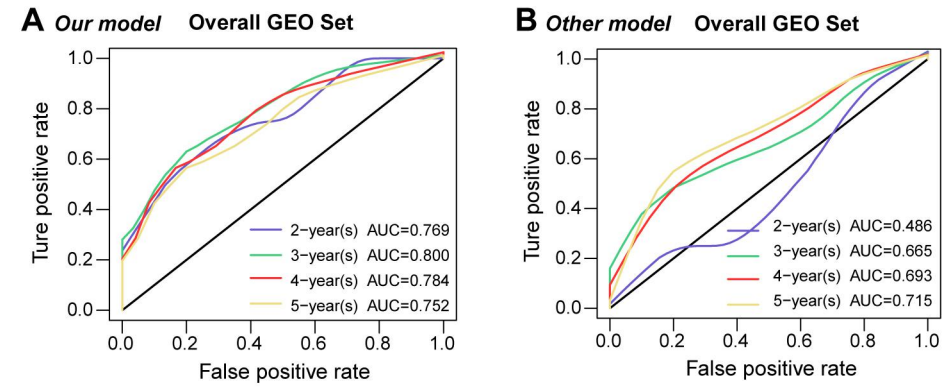

Figure S5: The ROC curves.

Supplement: Supplementary Materials — Figures S1 and S2: the survival analysis of DNMT1, DNMT3A, DNMT3B, MBD1, MBD2, MBD3, MBD4, MECP2, NEIL1, NTHL1, SMUG1, TDG, TET1, TET2, TET3, UHRF1, UHRF2, UNG, ZBTB4, and ZBTB33. Figure S3: the univariate analysis results of 54 genes. Univariate analysis showed that these 54 m5C-related genes might affect the survival time of the patients. Figure S4: the survival analysis of the m5C score in GSE39058. Figure S5: the ROC curves of our model (A) and others (B). Figure S6: the immune cell infiltration analysis by the CIBERSORT algorithm. [file 1629318.f1.zip › 1629318.f1/Figure S5.pdf]
